# Optimization of DNA extraction from dental remains

**DOI:** 10.1002/elps.201900142

**Published:** 2019-05-29

**Authors:** Concetta Cafiero, Agnese Re, Egidio Stigliano, Ezio Bassotti, Rossana Moroni, Cristina Grippaudo

**Affiliations:** ^1^ Università Cattolica del Sacro Cuore‐Dental Institute Rome Italy; ^2^ Università Cattolica del Sacro Cuore‐Institute of Pathological Anatomy Rome Italy; ^3^ Fondazione Policlinico Universitario A. Gemelli IRCCS Rome Italy; ^4^ Università Cattolica del Sacro Cuore Rome Italy

**Keywords:** Dental remains, DNA extraction method, Forensic, Tooth decalcification, Tooth opening

## Abstract

Efficient DNA extraction procedures is a critical step involved in the process of successful DNA analysis of such samples. Various protocols have been devised for the genomic DNA extraction from human tissues and forensic stains, such as dental tissue that is the skeletal part that better preserves DNA over time. However DNA recovery is low and protocols require labor‐intensive and time‐consuming step prior to isolating genetic material. Herein, we describe an extremely fast procedure of DNA extraction from teeth compared to classical method. Sixteen teeth of 100‐year‐old human remains were divided into two groups of 8 teeth and we compared DNA yield, in term of quantity and quality, starting from two different sample preparation steps. Specifically, teeth of group 1 were treated with a classic technique based on several steps of pulverization and decalcification, while teeth of group 2 were processed following a new procedure to withdraw dental pulp. In the next phase, the samples of both group underwent the same procedure of extraction, quantification and DNA profile analysis. Our findings provide an alternative protocol to obtain a higher amount of good quality DNA in a fast time procedure, helpful for forensic and anthropological studies.

Molecular biology techniques can be applied in forensic medicine to deny or confirm the source of ancient bone remains from an archeological site and verify the unknown identity of an individual 1, 2, 3, 4. In the early onset of the post‐mortem phase, blood and bodily fluids decompose, which lead to DNA degradation beginning with the release of endogenous intracellular enzymes (such as lipases, nucleases, and proteases). Teeth, on the other hand, are a skeletal structure that better preserves DNA over time. Due to their anatomy, with a naturally hard mineral composition and low porosity, teeth are more resistant to contamination compared to bone.

However, environmental contaminants (i.e., humic acid, fulvic acid, and metals), as well as microorganisms (i.e., bacteria or fungi), can have a negative impact on DNA extraction and, its amplification and analysis 5, 6, 7, 8, 9, 10. In addition, it can be noted that exogenous DNA is often less degraded when compared to endogenous DNA and can interfere with genetic analysis, resulting in erroneous DNA profiles. For these reasons, achieving higher quality in DNA extraction is still challenging.

Before extracting DNA from teeth, the possibility of coextracting factors that could alter the isolated nucleic acids and inhibit the Polymerase Chain Reaction (PCR) procedure should be considered, although similar methods are used in clinical practice for hard tissue samples 11, 12, 13.

“Classical” methods of DNA extraction from teeth include steps of pulverization and subsequent decalcification in several days. Therefore, PCR inhibition can be caused by components naturally present in teeth, such as calcium and collagen, and/or by some products used in the extraction process, such as ethylenediaminetetraacetic acid (EDTA), phenol, chloroform, sodium chloride (NaCl), and detergents 14. In addition, it is necessary to descale a sample before extraction. Currently, DNA extraction from teeth follows various steps, and it is a very long procedure that may negatively affect the extraction itself, even if the DNA molecules are highly stable under extreme conditions but are easily degraded by pH values far from neutrality 9.

With this knowledge, herein, we describe a new preparation method for DNA extraction from teeth compared to the current (and standard) method. Our procedure avoids the problems described above and allows a higher quantity of quality DNA; additionally, said procedure can be done hastily, which is critical in the forensic and legal fields.

The project of developing a new technique is due to the observation that when the teeth are opened without mixing different tissues (enamel, dentin, and pulp), the pulp tissue is easier to isolate and it is better preserved. The hypothesis is that DNA can be collected quickly and the risk of contamination is low.

The new technique has been tested on teeth obtained from the maxillary and mandibular bones of the same skull that was almost 100‐years old. The skeletal remains were kept in a calcareous stone of an ossuary.

A total of *N* = 16 teeth were taken from a single skull. The first group (denoted with Group 1 in the following) of *N*old = 8 teeth have been analyzed using the old method and the second group (denoted with Group 2 in the rest of the work) of *N*new = 8 teeth have been analyzed using the new technique.

It has been decided to consider *N* = 16 teeth from a single skull in order to remove the variation coming from several different skulls. In this way it is possible to assume that the only variation is the one coming from the two techniques. The skull was selected because it featured a complete and well preserved dentition. The teeth analyzed were: one incisor, one canine, and two premolars in each side of the upper and lower dental arches. Each tooth of the right side was compared to the homologue of the left one.

The lab procedures were conducted in sterile conditions and at room temperature (20–25°C) in order to avoid both contamination of exogenous DNA and degradation of the DNA of interest. All teeth were 1) observed under white light; 2) cleaned in a 6% sodium hypochlorite solution for 18 h; 3) washed first in a saline solution for 30 min, and then washed with psoralen; 4) exposed to ultraviolet (UVA) radiation at 254 nm wavelength for 30 min, to destroy superficial nucleic acids (from contaminants).

Thereafter, the teeth were divided into two groups of eight teeth per group: group 1 underwent the standard technique consisting of several steps of pulverization and decalcification before subsequent DNA extraction, whereas group 2 underwent the new swift preparation procedure, followed by the same DNA extraction procedure.

Group 1 received the preparation with standard procedure. Briefly, each tooth was pulverized using a Teflon pestle in a ceramic mortar. During the decalcification process, the resulting powder was dissolved in 12 mL of 0.5 M Tris‐HCl/EDTA solution in a 15 mL tube. The mixture was kept under shaking for 4 days at room temperature in order to obtain the DNA from the mineral matrix. After the last day (day 4), the mixture was filtered and lysed.

Group 2 was prepared with the new procedure. Each tooth was observed under a Nikon SMZ‐745T stereomicroscope (Nikon Instruments S.p.A., Firenze, Italy) to determine the least damaged part of the tooth (Figure 1). In the apical‐coronal axis, a 1 mm deep and 1 mm wide furrow was cut with a carborundum disk (40 × 1 mm, Henry–Schein KruggS.r.L, Milano, Italy) using a sterile straight hand‐piece applied to a surgical micro‐motor (Intrasurg® 300, KaVo Dental GmbH, Biberachan der Riß, Germania). We used intermittent time intervals in order to avoid heating the teeth, with low revolutions per min (2500 rpm) and without cooling the water. Afterwards, the teeth were opened with a 4.7 mm bi‐bevel chisel and mead mallet with nylon ends (Hu‐Friedy Mfg. Co., LLC3232 N. Rockwell St. Chicago, IL 60618–5935), the pulp cavity was exposed along with part of the root canal (Figure 2 A–C) and collected in a 2 mL tube. The harvested pulp material was rehydrated and lysed with a solution of 500 µL GT Buffer and 40 µL of proteinase K solution (10 mg/mL). This mixture was incubated at 56°C for 4 h, mixed for 5 s in sample tubes every hour. After incubation the supernatant was transferred into a filter column and centrifuged at 14 000 rpm for 5 min to obtain a limpid solution for DNA extraction.

**Figure 1:**
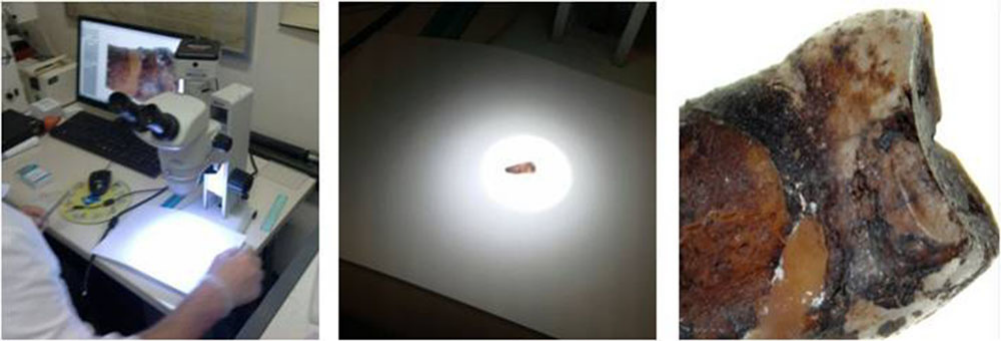
Representative image of teeth examination with a stereomicroscope before the clean up step. (Nikon Stereo‐microscope SMZ‐745T, Nikon Instruments S.p.A., Firenze, Italy – magnification 7X)

**Figure 2:**
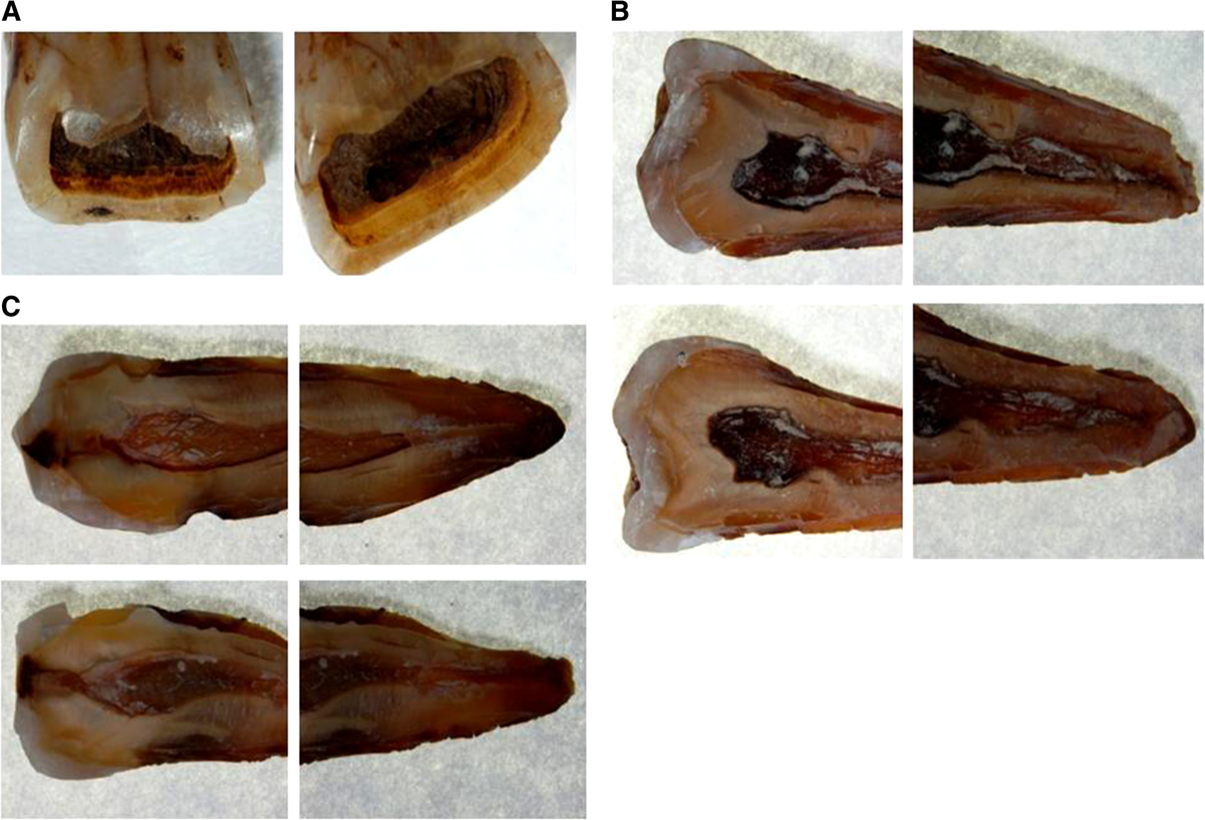
Representative image of teeth undergoing the new procedure extraction as described in the procedural steps section. A) Dentine; B) Opening and recovery of DNA in the pulp cavity and the root canal. C) Reticular channels. Images were taken with a stereomicroscope (Nikon Stereo‐microscope SMZ‐745T, Nikon Instruments S.p.A., Firenze, Italy – magnification 7X).

The solution of DNA collected from each tooth from group 1 and group 2 was processed separately by 401 MagCore® Genomic DNA Tissue Kit, using MagCore® Automated Nucleic Acid Extractor. DNA of groups 1 and 2 and relative blank was quantified using the Qbit 2.0 Fluorometer (Life technologies) according to manufacturer's instructions.

Table 1 shows the 16 DNA measurements resulting from the 16 teeth analyzed, divided in the two study groups.

**Table 1:** DNA measures coming from the 16 teeth and divided into the two already defined groups

	Total DNA ng/µL	
	Group 1	Group 2
	0.92	14.41
	0.90	17.58
	0.75	18.13
	0.98	16.54
	0.99	15.33
	0.95	14.20
	0.89	18.00
	0.85	16.50
Mean ± SD	0.905 ± 0.077	16.33 ± 1.553

A Shapiro‐Wilk test highlights the normality of both the distributions. Hence it is possible to compare the two means using the parametric *T* test for paired samples (Table 2) The paired sample *T* test produced a *p*‐value > 0.0001 therefore there is a significant difference between the two groups. In detail, it is possible to claim that group 2 presents better results in terms of extracted DNA quantity.

**Table 2:** Main measures of descriptive statistics

	Descriptive Statistics			
	Minimum	Maximum	Mean	SD
Group 1	0.753	0.993	0.90488	0.076911
Group 2	14.203	18.129	16.33650	1.552913

It is already evident that group 2 presents the highest amount of extracted DNA with a mean of 16.33 against group 1 with a mean of 0.905 (Table 3). Furthermore, to check DNA quality Short Tandem Repeat analyses were performed by GlobalFiler PCR Amplification Kit in DNA obtained from a positive control and a sample of Group 2 (Figure 3).

**Table 3:** Graphic presentation of the results, showing that group 2 presents the highest amount of extracted DNA with a mean of 16.33 against a group 1 with a mean of 0.905

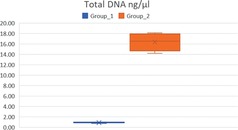


**Figure 3:**
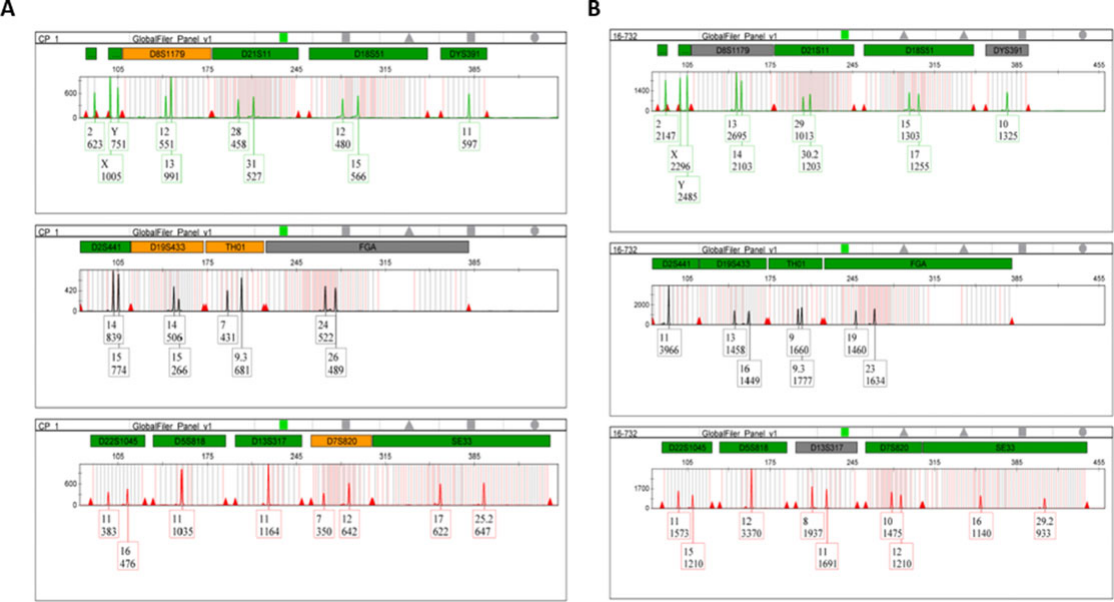
Representative Short Tandem Repeats (loci D8S1179, D21S11, D18S51, DYS391, D2S441, D19S433, TH01, FGA, D22S1045, D5S818, D13S317, D7S820 and SE33) profiles of DNA extracted from dental remain of a Control (A) and a sample of GROUP 2 (B). The fragment size is indicated below each peak.

In our experience, the technique based on direct tooth opening to collect dental paste from the endodontic system space without any chemical manipulation, displayed extremely advantageous results in terms of cost, time (from days to a few hours), quality, and quantity of DNA extracted; which, as previously mentioned, is extremely important in forensic and anthropological practice 15. Furthermore, this method can help reduce the contamination by exogenous DNA, which could give uncertain results in subsequent amplification techniques using PCR.

Many factors make DNA extraction from bone difficult, such as the presence of inhibiting factors that alter the conditions of nucleic acids or exogenous DNA contamination. DNA from bone remains may be often contaminated during handling before and/or after the extraction process; moreover bacterial or fungal DNA may contaminate most parts of the remains. The DNA of artifacts such as bones, teeth, and ancient tissues is typically degraded into small fragments of >300 bp, often only 50–200 bp. The extraction and subsequent amplification are therefore particularly difficult because methods currently used are not always satisfactory. In fact, one of the limits of the study of ancient findings is not being able to use a single reference method.

This study shows that the extraction yield was an average of 17.79 ± 2.93 greater when starting from direct extraction of pulp from the endodontic system space rather than the pulverized tooth, and that the extraction time was shortened and considerably reduced. This new method could be useful to improve DNA extraction in special cases such as ancient or badly preserved human remains, which in many situations, is a priority 16, 17, 18, 19.

*The authors have declared no conflict of interest*.
